# An Integrated Approach to Rapid Diagnosis of Tuberculosis and Multidrug Resistance Using Liquid Culture and Molecular Methods in Russia

**DOI:** 10.1371/journal.pone.0007129

**Published:** 2009-09-23

**Authors:** Yanina Balabanova, Francis Drobniewski, Vladyslav Nikolayevskyy, Annika Kruuner, Nadezhda Malomanova, Tatyana Simak, Nailya Ilyina, Svetlana Zakharova, Natalya Lebedeva, Heather L. Alexander, Rick O'Brien, Hojoon Sohn, Anastasia Shakhmistova, Ivan Fedorin

**Affiliations:** 1 National Mycobacterium Reference Laboratory, Institute of Cell and Molecular Sciences, Queen Mary College, Barts and the London School of Medicine, University of London, London, United Kingdom; 2 Samara Oblast Tuberculosis Dispensary, Samara, Russia; 3 Samara City Tuberculosis Service, Samara, Russia; 4 Foundation for Innovative New Diagnostic, Cointrin/Geneva, Switzerland; 5 US Centers for Disease Control and Prevention, Division of TB Elimination, Atlanta, Georgia, United States of America; University of Cape Town, South Africa

## Abstract

**Objective:**

To analyse the feasibility, cost and performance of rapid tuberculosis (TB) molecular and culture systems, in a high multidrug-resistant TB (MDR TB) middle-income region (Samara, Russia) and provide evidence for WHO policy change.

**Methods:**

Performance and cost evaluation was conducted to compare the BACTEC™ MGIT™ 960 system for culture and drug susceptibility testing (DST) and molecular systems for TB diagnosis, resistance to isoniazid and rifampin, and MDR TB identification compared to conventional Lowenstein-Jensen culture assays.

**Findings:**

698 consecutive patients (2487 sputum samples) with risk factors for drug-resistant tuberculosis were recruited. Overall *M. tuberculosis* complex culture positivity rates were 31.6% (787/2487) in MGIT and 27.1% (675/2487) in LJ (90.5% and 83.2% for smear-positive specimens). In total, 809 cultures of *M. tuberculosis* complex were isolated by any method. Median time to detection was 14 days for MGIT and 36 days for LJ (10 and 33 days for smear positive specimens) and indirect DST in MGIT took 9 days compared to 21 days on LJ. There was good concordance between DST on LJ and MGIT (96.8% for rifampin and 95.6% for isoniazid). Both molecular hybridization assay results correlated well with MGIT DST results, although molecular assays generally yielded higher rates of resistance (by approximately 3% for both isoniazid and rifampin).

**Conclusion:**

With effective planning and logistics, the MGIT 960 and molecular based methodologies can be successfully introduced into a reference laboratory setting in a middle incidence country. High rates of MDR TB in the Russian Federation make the introduction of such assays particularly useful.

## Introduction

Tuberculosis (TB) remains one of the leading causes of morbidity and mortality globally, focused principally, but not exclusively, in the non-industrialized world.

Timely diagnosis and prompt treatment of infectious cases are the key elements of the international effort to combat TB, providing cure of an individual patient and reducing the spread of TB by rendering infectious cases non-infectious.

Multidrug-resistant TB (MDR TB), i.e. resistance to at least isoniazid (Inh) and rifampin (Rif), and extensively drug-resistant TB (XDR TB), i.e. MDR plus resistance to amikacin, kanamycin or capreomycin and a fluoroquinolone, are the most problematic forms of resistance because treatment options are limited and the second-line drugs used for therapy are more toxic, less effective, more expensive, and must be administered for a longer period of time than standard first-line drug therapy [1].

The highest rates of MDR TB in the world (approximately 10% in new and 25% in re-treatment cases), have been reported from the Baltic region and countries of the former Soviet Union [2]–[11].

*Conventional culture and DST on solid media is a slow process, and in high income, low-incidence countries these systems have been supplemented (or replaced) by automated liquid culture systems such as the Becton Dickinson BACTEC™ MGIT™ 960 system. Decreased time to detection, greater sensitivity than Lowenstein-Jensen (LJ) solid media, comparable sensitivity to the radiometric Bactec 460 system in detecting* Mycobacteria *in clinical specimens, and good concordance with both LJ and Bactec 460 DST for first-line drugs (FLD) have been demonstrated in several studies [12]–[16].*

Rapid molecular methods, including commercial or in-house DNA hybridisation or amplification methods [17] allow detection of TB and rifampin resistance (and, for some, assays isoniazid resistance as well) in clinical samples within 1–2 days [18]–[25]. Despite the demonstrated advantages, the limited data on the performance, role and value of rapid culture, DST and molecular detection systems, together with concerns of increased cost and contamination rates relative to conventional culture on solid media, have dampened interest and progress in implementing these systems in low to middle income settings. However, this situation is changing in response to the growing MDR TB epidemic and the recent WHO recommendations on the use of liquid culture and DST and line probe assays (LPAs) [26], [27].

This study describes the feasibility of introduction, diagnostic accuracy and costs of the MGIT rapid culture system for primary specimens and FLD DST, coupled with rapid molecular systems for TB culture identification and detection of resistance to isoniazid and rifampin in Samara, Russia, a middle income region with a high burden of MDR TB [5], [9], [28]. The project was undertaken with the intention (achieved) of producing evidence for the implementation of global health policy changes relating to TB diagnosis by the WHO.

## Materials and Methods

### Ethics statement

The study was approved by the Samara Medical University Ethics Committee. The study received a waiver of informed consent because the study used samples that were routinely collected for use in approved routine tests on LJ media. The tests on the MGIT 960 system were performed in parallel with the approved routine tests on LJ media. All aspects related to culture and phenotypic DST were reviewed and approved by the U.S. Centers for Disease Control and Prevention as non-human subjects research which does not require informed consent.

### Setting and Design

A programmatic intervention and evaluation was conducted to compare the feasibility, utility and performance characteristics (recovery rates, time-to-detection) of the MGIT culture and DST system (Becton Dickinson, Sparks, MD) and rapid molecular systems to conventional standard reference LJ-based assays in patients at high risk of MDR TB in the central TB laboratory of Samara Region (Category 3 level facility), Russia Federation. The study was preceded by development of an agreed customer support plan that included installation and maintenance of the BACTEC™ machine as well as an uninterrupted supply of reagents needed based on reduced pricing policy offered for Samara. Principles outlined by the Standards for the Reporting of Diagnostic accuracy studies (STARD) for diagnostic accuracy studies were followed.

Prior to implementation into routine practice, MGIT and molecular methods were quality controlled and validated by the Health Protection Agency National Mycobacterium Reference Unit (HPA NMRL) according to the WHO/IUATLD Supranational Reference Laboratory (SRL) proficiency testing criteria [29] using a panel of defined *M. tuberculosis* cultures provided through the WHO SRL mechanism. Once the laboratory achieved pre-determined minimum standard efficiency levels based on WHO/IUATLD Supranational Reference Laboratory proficiency testing criteria for performance [29] of 80% (E and S), 89% (Inh), and 95% (Rif) in performing MGIT DST, MGIT and LJ culture and FLD DST were performed in parallel and all results were made available to clinicians. All staff was formally trained in bacteriological and molecular methods. Staff performing molecular assays was blinded to culture results and both were blinded to epidemiological data. This study also evaluated the detailed costs associated with the introduction of MGIT for TB culture diagnosis and DST in comparison with conventional methods on solid LJ media.

### Patient Population

Patients were enrolled from 8 TB clinics in Samara Oblast that are part of a specialised service which provides diagnostics and treatment for TB patients only. The clinics verify the diagnosis for patients referred by the general health care sector after initial screening with a high suspicion for TB. All confirmed or suspected pulmonary TB patients who were sputum smear-positive and/or at high risk for MDR TB (individuals with prior TB treatment, persistent smear-positivity after 3 months treatment and/or poor clinical improvement, relapse, default, repeated treatment interruptions, contacts of patients with confirmed MDR TB, homeless persons and former prisoners) were included.

Patients were excluded if they were currently receiving TB treatment and smear-converted or remained smear-negative, were known to be infected with an MDR TB strain, or were suspected of having extra-pulmonary TB without pulmonary involvement.

Enrolment commenced in April 2006 and continued through April 2008.

### Specimen preparation and primary culture

At least three routine sputum samples were collected from each patient into 50 ml screw-cap centrifuge tubes (Falcon, Becton Dickenson, USA) prior to treatment initiation as well as during treatment as follow-up control samples. Sputum samples were sent daily from the clinics to the laboratory; specimens were stored at 4°C until processed.

Specimens were processed using the NaOH-NALC method [30] employing the Becton Dickinson MycoPrep™ kit as described by the manufacturer. The final concentration of NaOH (1.0% w/v) was determined during the validation phase to maintain contamination rates below 8–10%. Concentrated specimens were stained for the presence of acid-fast bacilli (AFB) according to the Ziehl-Nielsen method [31].

All processed specimens were immediately inoculated on both MGIT (0.5 ml inoculum) and LJ media (0.2 ml inoculum).

The MGIT 960 was checked daily for positive and negative cultures and LJ cultures were checked at least weekly. Although this standard approach carried an observation bias for time to detection of positive cultures, it followed the accepted practice of periodic visual scanning of LJ slopes reported in all previous published studies of the MGIT and comparable systems and LJ culture. Tubes flagged as positive by the MGIT 960 instrument were examined visually for potential mycobacterial growth and growth was inoculated onto a blood agar plate, subcultured on an LJ slant and MGIT for DST, and an AFB smear was prepared.

### Isolate identification

Cultures were identified as *M. tuberculosis* complex (MTC) according to colony morphology, microscopic appearance, and standard biochemical assays, as specified in the Russian Federal guidelines [31].

Molecular tests also were used to decrease the time to identification of MTC. Previous studies suggested that liquid culture systems would increase the isolation of non-tuberculous mycobacteria (NTM) as well as *M. tuberculosis* [32]. Therefore, 327 consecutive mycobacterial cultures initially isolated on the MGIT 960 system were identified to species level using the GenoType® CM assay (Hain Lifesciences GmbH, Nehren, Germany).

### First Line drug susceptibility (DST)

The drug concentrations used in the MGIT system were (µg/ml): streptomycin (S): 1.0; isoniazid (Inh): 0.1; rifampin (Rif): 1.0; ethambutol (E): 5.0; pyrazinamide (Z): 100.0 [31], [33]. DST on LJ was performed according to the absolute concentration method, utilizing the following drug concentrations (µg/ml): S: 10.0; Inh: 1.0; Rif: 40.0; E: 2.0 [31]. Sensitivity to pyrazinamide is not routinely tested on LJ media according to Russian Federal guidelines [31].

As the majority of isolates were MTC (see Results), subsequent cultures were identified using an in-house macroarray, as described elsewhere [34] and/or a commercial “line probe” assay system for MTC identification and rifampin and isoniazid resistance (GenoType® MTBDR*plus*, Hain Lifesciences GmbH, Germany). Both systems employed the same basic principles i.e. polymerase chain reaction (PCR) amplification of relevant regions of genes including the *katG* and *rpoB* genes and *inhA* promoter region, followed by reverse phase hybridisation to probes immobilised on a solid phase membrane.

### Cost analysis

All laboratory procedures for both LJ and MGIT culture and DST were broken down into their component parts and a detailed time-and-motion study was conducted [35]–[37]. Total salaries, consumables costs, and capital (including equipment) infrastructure costs, maintenance, administrative and overhead costs of the laboratory, as well as transport costs were included in the final analysis.

Prices were converted into US dollars (USD) for this analysis. International pricings for all relevant laboratory resources and consumables for our study were based on published manufacturer suggested retail prices (MSRP) in developed countries such as the US. For local price analysis, procurement pricings specific to Samara with exceptions to MGIT instrumentation and consumables (for which we used the FIND-Becton Dickinson (BD) negotiated price available in 2006) were used. The usage of equipment, reagents, and laboratory space were quantified as minute used per square meter of space and minutes used. Overhead costs were calculated and allocated based on time-observation data particular to building space and staff utilization for each laboratory procedures included in our cost analysis.

### Statistical analysis

All data were obtained from records collected by the clinical and laboratory staff and entered in a password protected stand-alone database to maintain confidentiality.

Statistical analysis was performed using the SPSS version 15 package (SPSS, http://www.spss.com). The difference between rates among different groups was assessed using chi-test (χ^2^).

## Results

In total, 698 consecutive patients were recruited into the study and 2545 sputum samples were subjected to bacteriological examination on both LJ and MGIT media.

Initially, the MGIT 960 system yielded increased rates of culture contamination but rates were quickly lowered to 3.4% by meticulous adherence to the manufacturer's manual and protocols, and with rapid transport and/or refrigeration of samples. The samples collected from patients were immediately refrigerated and stored at +4 for a maximum time of 48 hours prior to decontamination and culture. All samples from participating study sites were transported in cool bags. Sterility checks of water, buffer and NALC solutions and disposables consumables (such as washes from sputum containers, cryovials and laboratory tubes used) were regularly run using blood agar plates. Negative controls of each batch of MGIT tubes and daily logs of all ready-made solutions were used to monitor any potential manufacturer's contamination. In order for any increased culture speed to be valuable, a rapid molecular identification method was essential to identify culture growth in 1 day; this also permitted TB identification in bacterially-contaminated cultures (data not shown). A proportion of cultures was also spoligotyped to exclude cross-contamination within the laboratory.

**Figure 1:**
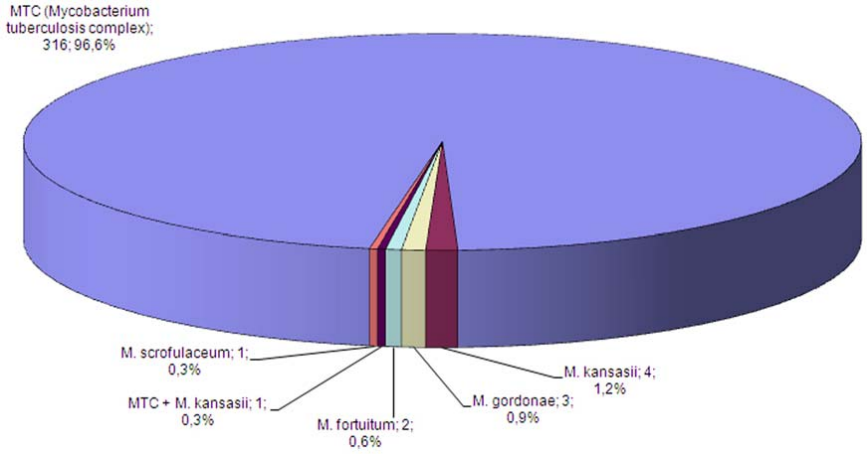
Mycobacterial speciation of cultures isolated from sputum specimens.

Of the first 327 consecutive patients with positive mycobacterial isolates, the applied GenoType® Mycobacterium CM assay demonstrated that the vast majority (96.6%) of isolates were *M. tuberculosis* complex (Figure 1). Subsequently, molecular methods were used to test all subsequent isolates simply for the presence or absence of MTC. Since very few NTMs were isolated, this paper presents results for MTC only. The overall MTC culture positivity rate for MGIT and LJ was 31.6% and 27.1% respectively (χ*^2^ = *11.9, p = 0.001); for smear positive specimens it was 90.5% and 83.2% (χ*^2^ = *8.6, p = 0.003) and for smear negative specimens, 20.4% and 16.4% respectively (χ*^2^ = *10.7, p = 0.001) (Table 1).

**Table 1:** MGIT and LJ culture positive sputum specimens by microscopy result (MTC only)*.

Sputum smear status	MGIT pos, n, (%)	Contaminated,n (%)	Positive and contaminated, n(%)	LJ pos, n (%)	Contaminated,n (%)	Positive and contaminated, n(%)
Smear positive(n-399)	361 (90.5%)	4 (1.0%)	2 (0.5%)	332 (83.2%)	2 (0.5%)	0
Smear negative(n-2088)	426 (20.4%)	81 (3.9%)	11 (0.5%)	343 (16.4%)	8 (0.4%)	1 (0.05%)
Total (n-2487)	787 (31.6%)	85 (3.4%)	13 (0.5%)	675 (27.1%)	10 (0.4%)	1 (0.04%)

* specimens for which both MGIT and LJ results available

The overall proportion of the total MTC isolates (number confirmed positive cultures by MGIT or LJ/total number positive cultures by either method) which were positive by MGIT was 97.2% (786/809) compared to 81.1% (656/809) for LJ. Of all culture positive specimens, 99.2% of smear-positive and 95.5% of smear-negative specimens were positive by MGIT while LJ recovery rates were 90.9% for smear-positives and 73.2% for smear-negative specimens. The concordance of results between the two systems was high for isolating MTC (92.7%) (Table 2).

**Table 2:** Recovery rates of each method and concordance compared to all positive cultures (MTC only)*.

*Micro-scopy*	Recovery rates of each method compared to all positive cultures						Concordance of MGIT and LJ culture results							
	*Total N positive cultures*	*MGIT+*	*MGIT recovery rate (%)*	*LJ+*	*LJ recovery rate (%)*	*χ^2^, p*	*MGIT+ and LJ+*	*MGIT- and LJ-*	*N concor-dant*	*Concor-dant (%)*	*MGIT+ but LJ-*	*MGIT- but LJ+*	*N discor-dant*	*Discor-dant (%)*
*formula*	*a = d+f+g*	*b*	*b/a*	*c*	*c/a*		*d*	*e*	*d+e*	*(d+e)/n*	*f*	*g*	*f+g*	*(f+g)/n*
Smear+ (n-393)	362	359	99.2%	329	90.9%	24.6, <0.001	326	31	357	90.8%	33	3	36	9.2%
Smear- (n-2003)	447	427	95.5%	327	73.2%	83.0, <0.001	307	1556	1863	93.0%	120	20	140	7.0%
Total(n-2396)	809	786	97.2%	656	81.1%	106.1, <0.001	633	1587	2220	92.7%	153	23	176	7.3%

* contaminated and indeterminate results excluded, specimens for which both MGIT and LJ results available.

(+) positive test, (−) negative test.

**Figure 2:**
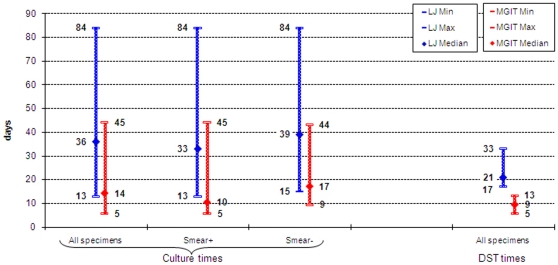
Time (days) to culture and DST results for mycobacterial cultures.

Among culture positives, the overall median time to detection of *M. tuberculosis* complex was 14 days and 36 days for MGIT and LJ, respectively. Indirect DST from isolates took an additional 9 days in MGIT and 21 days on LJ. Therefore, providing a rapid molecular identification method is available that takes 1–2 days to perform as in the case of the GenoType® MTBDR*plus* method and comparable methods such as in house *in situ* hybridisation methods [34], [38] the overall turn-around time can be as short as 25 days for MGIT vs approximately 60 days for LJ (Figure 2).

Comparative phenotypic DST data for both methods (Table 3) on all bacteriologically confirmed TB strains demonstrate approximately 63%, 50%, 27%, 60%, and 10% of the patients were resistant to isoniazid, rifampin, ethambutol, streptomycin, and pyrazinamide, respectively. Approximately 50% of cultures were MDR TB and nearly all rifampin-resistant isolates (98.7% and 100% detected by LJ and MGIT, respectively) were MDR TB. There was good concordance between the results obtained by the LJ and MGIT methods (Table 4) with agreement of 96.8% for rifampin, 95.6% for isoniazid but only 91.9% for ethambutol and for 89.5% for streptomycin.

**Table 3:** Phenotypic first-line DST comparing LJ and MGIT methodology.*

	*LJ*		*MGIT*	
	N	%	N	%
Total Patients with DST results (Inh+Rif)	319	100.0%	317	100.0%
Any resistance				
*Any resistance to isoniazid (Inh)*	195	61.1%	201	63.4%
*Any resistance to rifampin (Rif)*	158	49.5%	158	49.8%
*Any resistance ethambutol (E)*	81	25.4%	84	26.5%
*Any resistance to streptomycin (S)*	184	57.7%	192	60.6%
*Any resistance to pyrazinamide (Z)*			33	10.4%
Total Multi-Drug Resistance (MDR)	*156*	*49.8%*	*158*	*49.8%*
Total poly-resistance other than MDR	*36*	*11.3%*	*37*	*11.7%*
Total Susceptible	107	33.5%	104	32.6%

* DST was set up one culture per patient; contaminated and undetermined results excluded

**Table 4:** Comparative agreement of LJ and MGIT-based DST methods.

*DST*	No of concordant resistant	Total No resistant any methods	% concor-dance	No of concordant sensitive	Total No sensitive any methods	% concor-dance	Total agreement (sensitive and resistant)%
Inh (n-315)	190	197	96.4%	111	118	94.1%	95.6%
Rif (n-313)	153	158	96.8%	150	155	96.8%	96.8%
E (n-321)	76	89	85.4%	219	232	94.4%	91.9%
S (n-325)	176	193	91.2%	115	132	87.1%	89.5%

* for cultures, on which DST results were available from both methods; 11 MGIT and 9 LJ subcultures were contaminated across all four drugs.

The overall Inh, Rif and MDR resistance rates in the population as determined by the in-house macroarray (65.7%, 54.6% and 51.0% respectively) and the Hain methods (66.6%, 54.8% and 53.2% respectively) were comparable.

**There was good concordance for isoniazid and rifampin resistance between the commercial and in-house low-density array-based methods (88.5% and 80.7% for macroarray and 87.3% and 77.9% for Hain; 87.6% and 84.9% for macroarray and 84.4% and 82.2% for Hain respectively) compared with the MGIT culture or LJ systems The sensitivity and specificity of both methods when compared to either culture system were high: almost 93% and 94% for detection of isoniazid and approximately 87% and 94% for detection of rifampicin resistance against the MGIT system; approximately 92% and 93% for isoniazid and 90% and 93% for rifampicin against the LJ system respectively (Tables 5–6).**

**Table 5:** Comparison of molecular methods with MGIT culture results for isoniazid and rifampin resistance.

		*No concordant R^1^/Total No R by any method (%)*	*No concordant S^2^/Total No S by any method (%)*	*Total agree-ment^3^(S & R) %*	*Sensitivity^4^, %*	*Specificity^5^, %*	*Positive predictive value^6^, %*	*Negative predictive value^7^, %*
MA (n-305)	Inh	**188/207 (90.8%)**	**97/115 (84.3%)**	**88.5%**	**93.5%**	93.5%	**96.7%**	87.8%
	Rif	**140/177 (79.1%)**	**124/150 (82.7%)**	**80.7%**	**87.8%**	94.2%	**95.0%**	86.2%
Hain (n-311)	Inh	**193/214 (90.2%)**	**97/118 (82.2%)**	**87.3%**	**93.1%**	93.5%	**96.6%**	87.0%
	Rif	**148/197 (75.1%)**	**131/161 (81.4%)**	**77.9%**	**86.5%**	94.4%	**95.1%**	84.9%

**MA-macroarray method; Hain - GenoType® MTBDR*plus* method (Hain Lifesciences GmbH, Germany).**

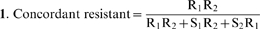
 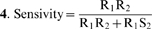 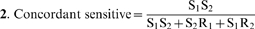 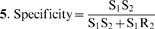 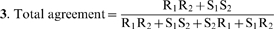 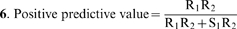 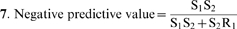

Where R-resistant and S-sensitive, 1 – by the test method (Hain or Macroarray), 2 – by the reference method (MGIT).

**Table 6:** Comparison of molecular methods with LJ culture results for isoniazid and rifampin resistance.

		*No concordant R^1^/Total No R by any method (%)*	*No concordant S^2^/Total No S by any method (%)*	*Total agree-ment^3^(S & R) %*	*Sensitivity^4^, %*	*Specificity^5^, %*	*Positive predictive value^6^, %*	*Negative predictive value^7^, %*
MA (n-305)	Inh	**195/216 (90.3%)**	**101/122 (82.8%)**	**87.6%**	**92.9%**	**94.4%**	**97.0%**	**87.1%**
	Rif	**152/177 (85.9%)**	**129/154 (83.8%)**	**84.9%**	**90.5%**	**93.5%**	**94.4%**	**89.0%**
Hain (n-311)	Inh	**194/221 (87.8%)**	**99/126 (78.6%)**	**84.4%**	**91.1%**	**92.5%**	**96.0%**	**83.9%**
	Rif	**154/185 (83.2%)**	**133/164 (81.1%)**	**82.2%**	**88.0%**	**93.0%**	**93.9%**	**86.4%**

**MA-macroarray method; Hain - GenoType® MTBDR*plus* method (Hain Lifesciences GmbH, Germany).**

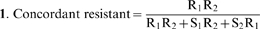
 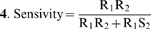 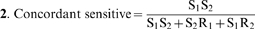 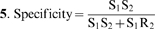 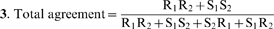 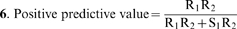 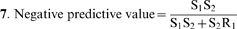

Where R-resistant and S-sensitive, 1 – by the test method (Hain or Macroarray), 2 – by the reference method (LJ).

Compared to the LJ method, MGIT culture was consistently more expensive than LJ regardless of pricing levels but the difference was small (Table 7). However, the FIND-BD pricing agreement brings about a 40% reduction in overall costs for screening one specimen for full first line DST with MGIT at $32 as compared to $56 for full catalogue pricing. LJ methodology costs ranged from $17 to $20 in Samara and international prices; if only isoniazid and rifampin resistance was tested the equivalent costs for Samara and internationally were $13 and $15 for LJ and $17 and $28 for the MGIT system.

**Table 7:** Comparison of overall costs for primary screening, first line DST testing per specimen screened for MGIT, LJ and molecular identification (Macroarray) tests (US dollar).

Pricing and resource input	Prima-ry culture				FLD DST (SIRE)				FLD DST (IR)				Macroarray^≠^
	MGIT Int'l	LJ Int'l	MGIT FIND-BD/Local	LJ Local	MGIT Int'l	LJ Int'l	MGITFIND-BD/Local	LJ Local	MGIT Int'l	LJ Int'l	MGIT FIND-BD/Local	LJ Local	
Decontamination*	3.32	3.32	2.76	2.76	3.32	3.32	2.76	2.76	3.32	3.32	2.76	2.76	NA
Prep LJ	N/A	0.22	N/A	0.10	N/A	1.08	N/A	0.49	–	0.65	–	0.29	NA
Test													
Overhead	1.94	4.95	1.94	4.95	3.42	7.93	3.42	7.93	1.37	5.97	1.37	5.97	1.57
Building	0.42	1.07	0.42	1.07	0.54	1.48	0.54	1.48	0.42	1.18	0.22	1.18	0.01
Equipment	1.92	0.50	1.45	0.24	4.69	2.69	2.45	1.11	2.40	1.35	1.21	0.66	0.22
Staff	0.51	1.34	0.51	1.34	1.66	2.96	1.66	2.96	0.66	2.02	0.66	2.02	1.66
Medical supplies	5.08	0.09	4.58	0.05	42.04	0.36	21.58	0.28	19.88	0.35	10.50	0.58	5.20
Sub-total Test	9.87	7.95	8.90	7.66	52.35	15.42	29.66	13.76	24.40	10.87	13.96	10.42	12.73
Total	13.74	11.49	11.66	10.50	55.68	19.83	32.42	17.00	27.73	14.84	16.71	13.47	14.30

* includes specimen transportation costs.

**≠** Macroarray steps include all steps from DNA extraction to reading/reporting of the results.

Int'L – international prices.

NA – not applicable.

The cost of performing the in-house method (macroarray) in Samara was calculated based on local wages and overhead costs: the overall average unit cost of the macroarray test was at about $15 per specimen. The total chemical and reagent components of the test was between $5–6 per specimen (Table 6). The test-strips used for this method were produced at the HPA MRU, London; more detailed assessment of the total assay costs when produced in Russia is needed and is subject of another on-going study.

The costs of the GenoType® MTBDR*plus* assay were not assessed within this project.

## Discussion

This study describes the performance characteristics of rapid liquid culture (MGIT 960) and molecular assays for the identification of MTC, rifampin, isoniazid and MDR TB as well as costs of the MGIT 960 system when introduced into Samara, a region within the Russian Federation with high rates of drug resistance and MDR TB [11].

The MGIT 960 method was quickly taken up by the staff and successfully introduced into practice in an escalating manner from primary culture to DST. An initially high contamination rate was lowered to 3.4% within a month of initiation of the project by meticulous adherence to manufacturer's instructions, use of standard protocols as well as a well-developed system of rapid sample collection and transport logistics. Coupled with high recovery rates, it demonstrates that decontamination procedure was not overly harsh and permitted adequate growth of mycobacteria while ensuring low contamination rates. A preliminary analysis presented here found that over 96% of positive cultures were *M. tuberculosis* complex, suggesting that frequent NTM isolation (as reported elsewhere [32]) was unlikely to be a significant problem in this study population. This was probably due to the high proportion of smear-positivity and drug resistant TB among enrolled patients, lower HIV-positivity rates compared to African countries, and dominance of the Beijing family TB strains in the area. This strain family has been actively transmitted in the area and has a strong association with drug resistance [5]. Subsequent culture growth was identified using a second commercial rapid identification system and a non-commercial in house system for MTC, isoniazid and rifampin resistance which both employed the same principle of PCR amplification coupled with a reverse phase hybridisation detection system.

As reported in high-resource, low-TB incidence settings, a greater proportion of positive cultures from primary specimens grew in the MGIT system primarily due to increased culture sensitivity for smear-negative specimens.

The median time for culture isolation was significantly faster for the MGIT 960 compared to LJ at 14 days versus 36 days for all specimens in agreement with other international studies mainly from low incidence [32], [39]–[42].

The proportion of drug resistance was very high in the studied population – almost half of the isolates were MDR TB. Mono –resistance to rifampicin was very rarely seen and nearly all rifampin-resistant isolates were MDR TB suggesting that rifampin resistance may serve as a reliable surrogate marker for MDR TB in this population. The median time to obtain DST results from positive cultures was faster with the MGIT system at 9 versus 21 days for LJ based methods in line with previously published works mainly from countries with lower levels of drug resistance [43]. Therefore introduction of the MGIT method can significantly decrease the overall turn-around time to 25 days comparable to data reported elsewhere [44]). However, within the project framework the turn-around-time for positive cultures and DST ranged from 13 to 87 days with a median of 38 days for the MGIT. Delays occurred due to logistical problems during the introduction of the MGIT system into routine use (e.g. reagents supply), training of additional personnel, contamination and delays between receiving a culture and subculturing for DST. One of the advantages of using the MGIT system was an opportunity to reliably determine sensitivity to pyrazinamide. This test is not routinely performed on LJ media in Russia due to the lack of standard protocols and variable standardization recommended on the national level.Although the MGIT system generated all FLD DST results more rapidly than the LJ methodology, the molecular methods provided results for isoniazid and rifampin resistance within one day. Another study performed in the same setting of the Samara Regional Tuberculosis Laboratory presented evidence for efficient use of molecular assays (GenoType® MTBDRplus) directly on smear-positive sputum samples [45]. The current study demonstrated that concordance of the commercial and in-house molecular methods for isoniazid and rifampin resistance was high, with very close but not complete agreement for isoniazid and rifampin resistance between the molecular and MGIT defined DST results. These methods could be implemented as an initial screen for MDR TB (directly on smear-positive samples or on mycobacterial cultures isolated from smear-negative samples), permitting the institution of infection control measures at an earlier stage, as well as more rapid provision of appropriate treatment in line with recent WHO recommendations that were developed with the support of the Samara project data [26]. The presence of mutations indicating resistance could be used as an indicator for simultaneously initiating first- and second-line resistance testing in MGIT, which could significantly reduce the delay in administering an appropriate drug regimen to an MDR TB (or XDR TB) case.

The economic analysis demonstrated that although the MGIT culture system was slightly more expensive than the LJ method ($12 versus $11 respectively), it would permit earlier diagnosis of TB and prompt treatment initiation (a reduction in median culture time of 22 days).

Similarly MGIT FLD DST was more expensive than LJ FLD DST ($56 versus $20 using international prices) but the difference allowed a significant decrease in diagnostic turn-around time resulting in earlier identification of drug resistance, including MDR TB, especially when FLD and SLD DST are set up simultaneously for isolates which were diagnosed as having mutations to rifampicin and isoniazid by molecular methods. Coupled with molecular systems for rapid identification and drug resistance detection, this would have a significant impact on a timely administration of an adequate treatment regimen and potentially improve treatment outcomes. Administration of timely therapy will render an individual non-infectious and interrupt transmission; molecular methods and/or MGIT based DST identify the many patients who have MDR TB (who will not be rendered non-infectious by standard TB therapy and so will continue to transmit MDR TB) and culture –based phenotypic methods are the only way of reliably identifying the antimicrobials that are able to render MDR TB individuals non-infectious.

This demonstration project provided much of the evidence underpinning the diagnostic policy changes relating to bacteriological culture adopted by the WHO in 2007-8. Currently WHO recommend the routine use of TB liquid culture and DST even in resource-limited settings to improve diagnosis of TB in general, MDR TB and smear-negative pulmonary TB including application of a rapid method of species identification [27]. The higher cost of the automated liquid culture media systems is currently being addressed by the manufacturers by introducing changes into the pricing policy for the public sector in lower income countries. This project showed that it is possible to successfully introduce this technology into resource-constrained settings but that to achieve satisfactory implementation and performance of the MGIT system (which is more prone to bacterial contamination due to the greater sensitivity of liquid media for culture of mycobacteria as well as other microorganisms compared with solid culture and for DST which is more complex to perform that using solid culture) key issues needed to be resolved. These include: (1) availability of appropriate Category 3 level laboratory infrastructure including an agreed maintenance plan for the BACTEC system; (2) repetitive on-site training of laboratory personnel in MGIT methodology (using detailed SOPs and the system manual) and molecular methods to create a multi-skilled cadre of staff; (3) initial expert observation of the performance and implementation of internal and external quality control of laboratory work; (4) development of effective logistics for timely collection, storage and transport of fresh sputum samples to the laboratory as well as the reporting of results; (5) the creation of algorithms for laboratory work flow and computerized laboratory record keeping; (6) timely maintenance of equipment and ensuring a safe continuous supply of reagents by establishing a commercial contract with a manufacturer and (7) introduction of a robust stock control system.

For these diagnostic culture systems to have a maximum therapeutic impact there must be rapid identification of cultures with the ability to analyse first and second-line DST phenotypically when molecular tests demonstrate the presence of mutations encoding rifampin (and possibly isoniazid) resistance in the original patient specimen or the resulting culture. This will significantly reduce the time between sputum collection and full susceptibility testing for MDR TB cases. Addressing timeliness in technological improvement should go in tandem with minimizing organizational delay. Clinicians need to make prompt therapeutic changes following rapid DST analysis.

With effective planning and logistics, an adequate decontamination protocol and careful training, the MGIT 960 and molecular-based methodologies can be successfully introduced into a reference laboratory setting in a middle TB incidence country. The high rates of MDR TB in the Russian Federation make the introduction of such assays particularly useful and are likely to translate to other settings with a high level of drug resistance or where the additional speed of diagnosis and increased diagnostic sensitivity are of value as in HIV associated tuberculosis.
